# Keloid of Pinna Revealed a Chondroid Syringoma in Disguise

**DOI:** 10.7759/cureus.62060

**Published:** 2024-06-10

**Authors:** Gopikrishnan Vijayakumar, Anju Devi, Anjali Narwal, Mala Kamboj

**Affiliations:** 1 Oral Pathology and Microbiology, Pandit Bhagwat Dayal Sharma Post Graduate Institute of Dental Sciences, Rohtak, IND

**Keywords:** apocrine tumor, keloid, mixed tumour, pleomorphic adenoma, chondroid syringoma

## Abstract

Cutaneous mixed tumor or chondroid syringoma is a rare benign, skin appendageal tumor prevalent in areas of the head and neck. It represents the cutaneous counterpart of the pleomorphic adenoma of salivary glands. Its clinical presentation often misguides the clinician to underdiagnose it as a reactive lesion. We report the case of a 94-year-old male admitted for excision of cutaneous carcinoma concurrently with a chondroid syringoma of the pinna provisionally misdiagnosed as a keloid.

## Introduction

The cutaneous mixed tumor or chondroid syringoma (CS) is a rare skin appendageal benign neoplasm initially described in 1961 by Hirsch and Helwig [1]. It typically occurs in elderly patients with male preponderance, frequently in the head and neck region, and is considered a counterpart of mixed tumors of the salivary glands (pleomorphic adenoma) [2,3]. The common sites of occurrence include the nose, chin, scalp, cheek, upper lip, and forehead. This tumor is unique among benign skin tumors as the incidence is very low and CS on the pinna is extremely rare [3-5]. The clinical presentation alone is often insufficient for the diagnosis, which occurs following histological evaluation. This tumor can easily be confused with other common benign conditions like an epidermoid cyst, pilar cyst, neurofibroma, dermatofibroma, histiocytoma, implantation dermoid, and even a sebaceous cyst [1,4,5]. Though the lesion is usually benign, malignant chondroid syringoma has also been reported in the literature [3,6].

## Case presentation

**Figure 1:**
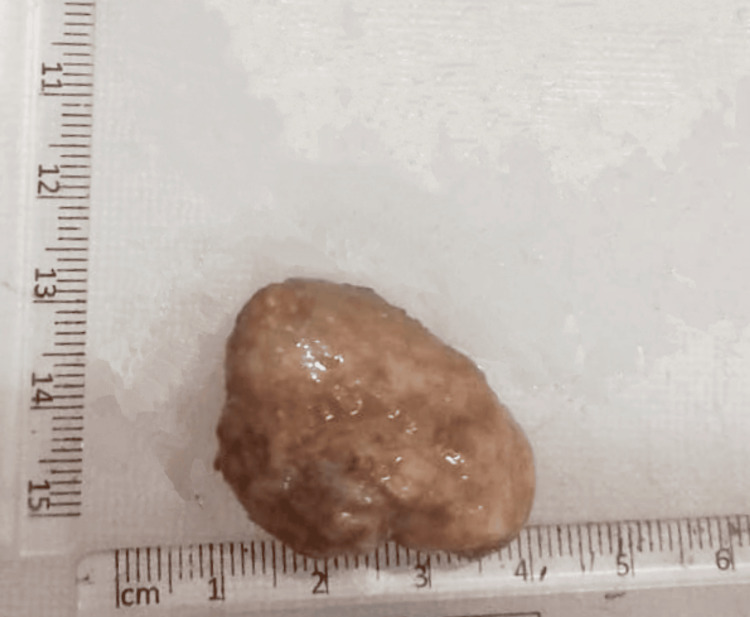
Gross specimen measuring 2.5 cm in dimension, greyish-nodular in appearance, firm to hard in consistency, submitted as a keloid from pinna. cm-Centimeter

A 94-year-old man admitted for surgical excision of cutaneous squamous cell carcinoma on his right cheek also had a 3 cm firm, painless, nodular mass over the left pinna. He reported that the lesion had been present for over two years and had grown slowly to date. On examination, the patient was moderately built with an Eastern Cooperative Oncology Group (ECOG) performance status score of three with no associated history of diabetes, hypertension, or other systemic illness. The mass was surgically excised along with the primary tumor of the cheek and was sent for histopathological analysis considering a provisional diagnosis of keloid. The mass was bony hard and appeared well-circumscribed, with a white tan on the gross (Figure 1).

**Figure 2:**
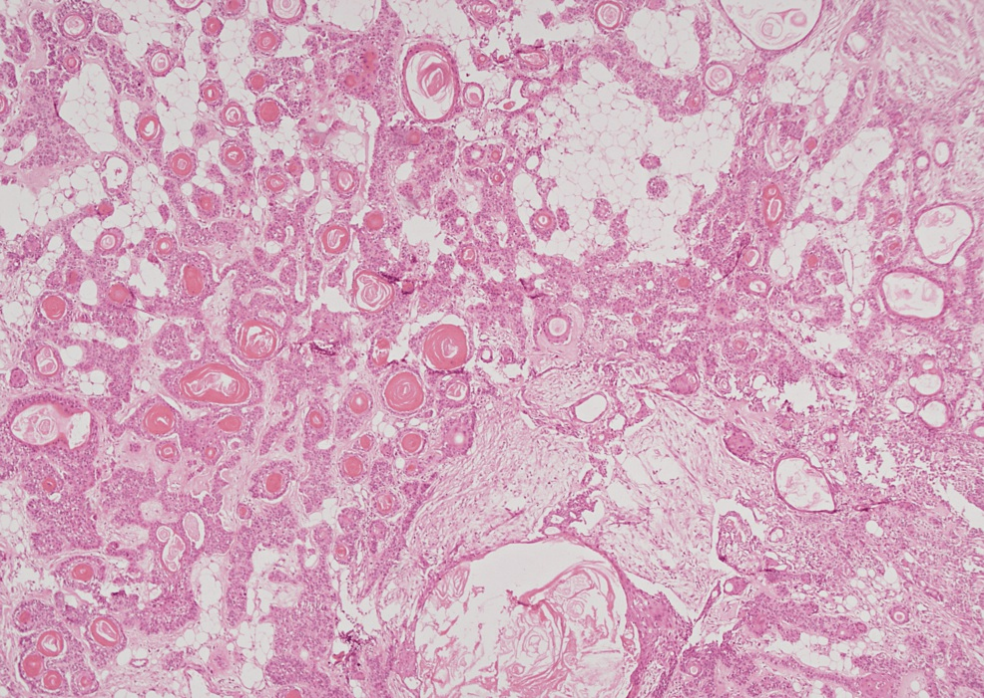
Photomicrograph showing dermal neoplasm with tumor cells arranged in cords and duct-like architecture. Numerous areas with follicular differentiation were seen as keratinous cysts (40X).

Multiple sections sampled and studied by histopathology demonstrated a well-circumscribed nodular dermal neoplasm with tumor cells arranged in cords and duct-like architecture. These ductal components had inner epithelial cells with round to oval nuclei, eosinophilic cytoplasm, and outer myoepithelial cells (Figure 2).

**Figure 3:**
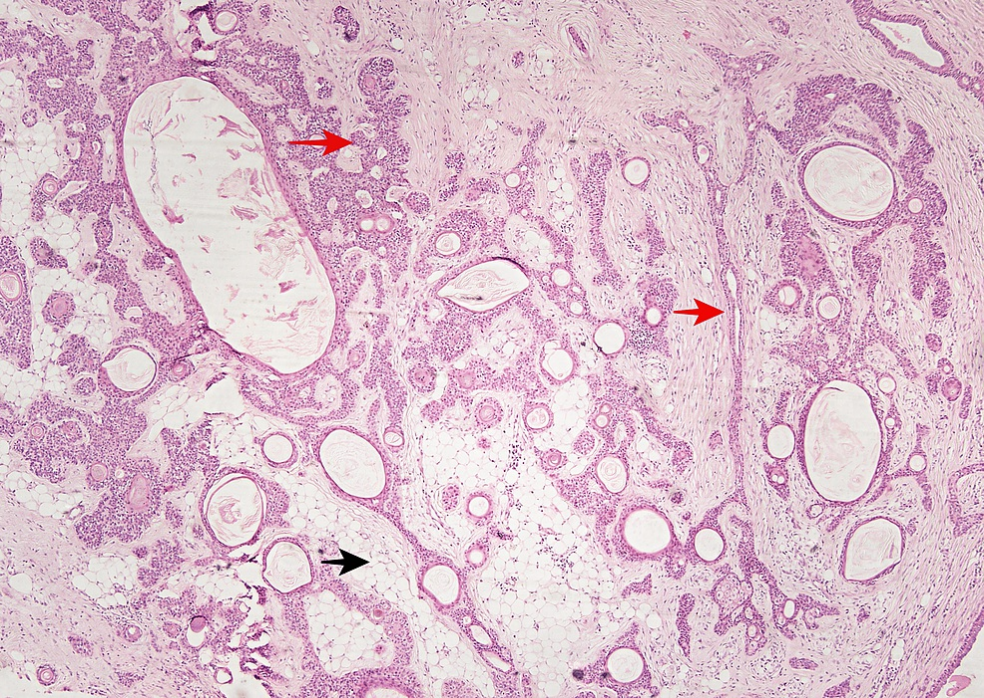
Photomicrograph showing elongated branching tubular structures (red arrow). Adipocytic metaplasia within the stroma (black arrow) (40X).

Numerous areas with follicular differentiation were seen as keratinous cysts with infundibular-type keratinization. The stroma was predominantly chondroid with few adipocytic metaplasia (Figure 3).

**Figure 4:**
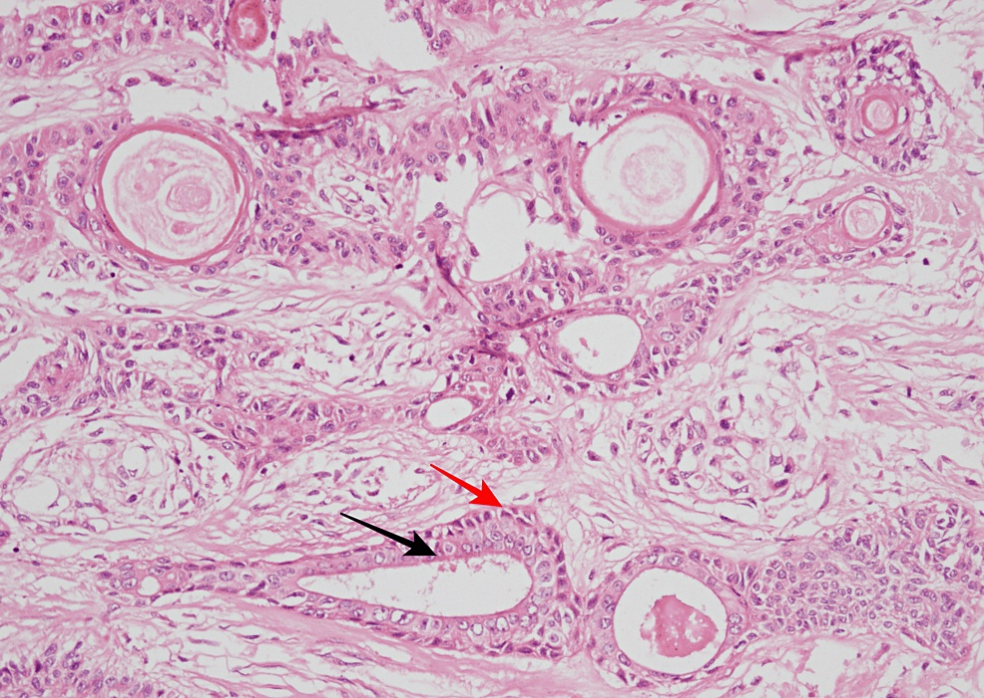
Photomicrograph showing two cell layered tubular structures with outer cuboidal (red arrow) and inner columnar cells (black arrow) (200X).

The irregular distribution of bilayered tubular structures is shown in Figure 4.

**Figure 5:**
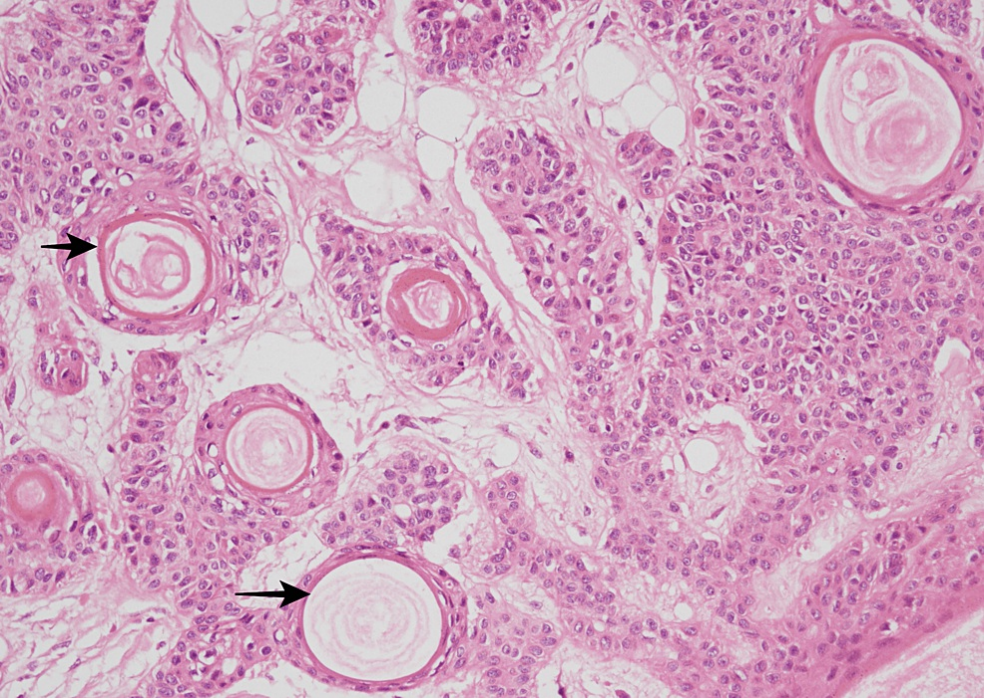
Photomicrograph showing follicular differentiation in the form of kerationous cyst with the infundibular type of keratinization (black arrow) (400X).

The follicular differentiation in the form of keratinous cysts with infundibular keratinization can be seen in Figure 5.

These favored the apocrine type of chondroid syringoma. No feature of malignancy was evident. A final diagnosis of chondroid syringoma was confirmed and reported. The primary tumor from the right cheek was diagnosed as moderately differentiated squamous cell carcinoma with a pathological staging of pT3 and Brigham and Women (BWH) tumor classification of T2a. The patient is under follow-up for the same.

## Discussion

Chondroid syringoma (CS)/cutaneous mixed tumor/ apocrine mixed tumor /eccrine mixed tumor or called ‘pleomorphic adenoma of sweat gland’ is a rare mixed skin tumor constituting 0.01%-0.1% of all primary skin tumors [1,2,7,8]. It was first described by Billroth while the term ‘chondroid syringoma’ was coined by Hirsch and Helwig [1,7]. They are more commonly found on the scalp, head, and neck regions and less frequently on the axilla, trunk, genitalia, and inguinal area [1]. Chondroid syringoma of the external auditory region is rare and often derives from the ceruminous glands in the skin of the external auditory canal [5]. Its occurrence at pinna is extremely rare, with a single case reported as malignant CS in literature [3]. CS as the name indicates the stroma is predominantly chondroid but also myxoid, fibrous, or osseous with epithelial structures typically apocrine or eccrine with pilosebaceous elements [1]. Five essential histological criteria have been proposed for the diagnosis of CS by Hirsch and Helwig namely, nests of cuboidal and polygonal cells, interconnecting tubular or alveolar structures lined by two or more cuboidal cell layers, ductal structures lined by one or two rows of cuboidal cells, occasional keratinous cysts and stroma of varying composition. Two histologic variants of CS have been described by Headington, namely the eccrine type comprising of small lumens, lined by a single layer of cuboidal epithelial cells with myxoid or chondroid stroma and apocrine type with irregular or haphazard arrangement of branching tubules and lumina lined by double rows of epithelial cells [4,9,10]. The stroma was predominantly chondroid occupying almost 75% of the tumor, while few focus showed adipocytic metaplasia. The resemblance of CS (pleomorphic adenoma of sweat glands)with pleomorphic adenoma of the salivary gland is commendable histomorphologically and on a molecular basis. Recent studies demonstrate similar chromosomal rearrangements of fusion genes encoding pleomorphic adenoma gene 1 (PLAG1) and the high‑mobility group AT‑hook 2 (HMGA2) protein in both tumors [9]. Malignant CS has been reported, and the histopathology of such shows cellular pleomorphism, hyperchromatic nuclei with atypia, infiltrative margins, necrosis, satellite nodules, and involvement of the adjacent structures [3,6]. The management of CS is predominantly surgical and consists of complete excision of the tumor.

## Conclusions

While diagnosing nodular or keloid-like lesions in the head and neck the chondroid syringoma could be considered in the differential diagnosis. Treatment is surgical excision with nearly no chance of recurrence however reports of malignant chondroid syringoma warrant early diagnosis and follow-up.

## References

[1] Khan K, Dinesh A, Landa M, Engdahl R (2019). A rare forehead mass: the chondroid syringoma. Cureus.

[2] Park SH, Kang SG, Choi HJ (2017). Chondroid syringoma of a cheek. J Craniofac Surg.

[3] Krishnamurthy A, Aggarwal N, Deen S, Majhi U, Ramshankar V (2015). Malignant chondroid syringoma of the pinna. Indian J Nucl Med.

[4] Reddy PB, Nandini DB, Sreedevi R, Deepak BS (2018). Benign chondroid syringoma affecting the upper lip: report of a rare case and review of literature. J Oral Maxillofac Pathol.

[5] Vasileiadis I, Kapetanakis S, Petousis A, Karakostas E, Simantirakis C (20112011). Rapidly growing chondroid syringoma of the external auditory canal: report of a rare case. Case Rep Med.

[6] Sekar R, Kalaiarasi R, Ganesan S, Alexander A, Saxena SK (2019). Malignant chondroid syringoma with nose and paranasal sinus extension: a case report. Allergy Rhinol (Providence).

[7] Walvekar PV, Jakati S, Bothra N, Kaliki S (2021). Isolated eyelid chondroid syringoma: a study of two cases. BMJ Case Rep.

[8] Nagano R, Fujii S, Wada H (2022). Lipomatous mixed tumor of the skin with cystic formation affecting the upper lip: a case report. Exp Ther Med.

[9] Gotoh S, Ntege EH, Nakasone T, Matayoshi A, Miyamoto S, Shimizu Y, Nakamura H (2022). Mixed tumour of the skin of the lower lip: a case report and review of the literature. Mol Clin Oncol.

[10] AlSaidan L, Sarkhouh M, Alenezi A, AlSabah H, Al Aradi I, Alterki A (2024). Chondroid syringoma of the nose: a rare case report and literature review. Int J Surg Case Rep.

